# Supplementary material for: Essential Oils from Colombian Plants: Antiviral Potential against Dengue Virus Based on Chemical Composition, In Vitro and In Silico Analyses
Source: Molecules. 2022 Oct 12;27(20):6844. doi: 10.3390/molecules27206844 (PMC9607004; doi:10.3390/molecules27206844)
Supplement: Supplementary file 1 [file molecules-27-06844-s001.zip › molecules-1919658-supplementary.pdf]

Supplementary Materials

Essential Oils from Colombian Plants: Antiviral Potential against Dengue Virus Based on Chemical Composition, In Vitro and In Silico Analyses

Lina Silva-Trujillo [1], Elizabeth Quintero-Rueda [1], Elena E. Stashenko [1], Sergio Conde-Ocazionez [2], Paola Rondón-Villarreal [2], and Raquel E. Ocazionez [1,*]

[1] Centro de Cromatografía y Espectrometría de Masas, CROM-MASS, Universidad Industrial de Santander, Bucaramanga 680002, Santander, Colombia. (LST); (EQR); (EES); (REO)

[2] Instituto de Investigación Masira, Facultad de Ciencias de la Salud, Universidad de Santander, Bucaramanga 680003, Santander, Colombia. (SCO); (PRV)

* Correspondence: Raquel E Ocazionez. relocaz@uis.edu.co

Table S1. Chemical composition of essential oils studied in this work

Compound	LRIs (DB-5MS column)		LaCi	LaCif	LaCaf1	LaCaf2	LoP	LoC	LoT	LoTf	LoTC	TdS1	TdS2	PaS1	ObS1	VcS1
	Exp.	Lit.														
cis-3-Hex-3-en-1-ol	853	850[a]	0.2	-	-	-	-	-	-	-	-	-	-	-	-	-
α-Thujene	927	930[a]	-	-	-	-	-	1.6	-	-	0.7	-	-	-	-	-
α-Pinene*	935	932[a]	0.3	0.2	0.2	-	1.7	0.4	-	-	0.4	-	-	4.6	0.5	9.4
Camphene*	951	954[a]	-	-	0.2	-	-	0.2	-	-	-	-	-	-	-	-
Sabinene	976	975[a]	-	-	-	-	-	-	-	-	-	-	-	-	0.5	0.6
Oct-1-en-3-ol	978	980[b]	0.2	-	0.004	-	-	-	-	-	-	-	-	-	-	-
β-Pinene*	982	974[a]	-	-	-	-	-	-	-	-	-	-	-	1.2	1.0	4.1
6-Methyl-hept-5-en-2-one*	985	985[a]	1.3	3.3	-	-	-	-	-	-	-	-	-	-	-	-
β-Myrcene*	990	990[a]	-	-	1.4	-	1.3	2.4	0.4	-	5.2	0.2	0.2	1.5	0.6	0.7
Octan-3-ol	994	991[a]	-	-	0.02	-	-	-	-	-	-	-	-	-	-	-
α-Phellandrene	1005	1002[a]	0.1	-	-	-	7.1	-	-	-	0.5	-	-	4.4	-	-
β-Phellandrene	1009	1002[a]	-	-	-	-	1.4	-	-	-	-	-	-	-	-	-
Δ^3-Carene	1012	1011[a]	-	-	-	-	0.7	0.1	-	-	0.3	0.2	-	0.6	-	-
α-Terpinene	1020	1017[a]	-	-	-	-	0.5	1.7	-	-	2.9	1.3	1.6	0.8	-	-
p-Cymene*	1027	1024[a]	-	-	-	-	12.6	14.4	2.3	2.0	19.1	3.0	3.6	3.0	-	-
Limonene*	1034	1029[a]	2.4	5.9	82.2	0.9	2.1	0.3	-	-	0.9	-	-	6.0	-	0.8
1,8-Cineol*	1036	1031[a]	-	-	-	-	13.0	1.3	-	-	-	-	-	3.6	8.1	0.8
cis-β-Ocimene	1046	1050[a]	-	-	-	-	-	-	-	-	1.2	-	-	-	-	-
trans-β-Ocimene	1047	1050[a]	-	0.4	1.0	-	-	-	-	-	0.4	-	-	0.4	0.3	-
γ-Terpinene	1061	1059[a]	-	-	-	-	2.4	5.3	0.9	6.9	9.2	0.6	0.7	0.8	-	-
cis-Sabinene hydrate	1072	1070[a]	-	-	-	-	-	0.3	-	-	-	-	-	-	-	-
Terpinolene	1088	1088[a]	-	-	-	-	-	-	-	-	0.3	-	-	0.8	-	-
α-Terpineol	1098	1088[a]	-	-	-	-	0.5	-	-	-	-	-	0.2	-	-	-
Linalool*	1099	1096[a]	1.1	2.5	0.4	0.5	0.7	1.0	-	-	0.3	-	-	0.4	42.7	-
trans-*p*-Mentha-2,8-dien-1-ol	1123	1122[a]	-	-	0.1	0.4	-	-	-	-	-	-	-	-	-	-
cis-p-Menth-2-en-1-ol	1129	1123[b]	-	-	-	-	-	-	-	-	-	-	-	0.5	-	-
cis-Limonene oxide	1141	1136[a]	-	-	0.3	0.1	-	-	-	-	-	-	-	-	-	-

Table S1. Cont.

Compound	LRIs (DB-5MS column)		LaCi	LaCif	LaCaf1	LaCaf2	LoP	LoC	LoT	LoTf	LoTC	TdS1	TdS2	PaS1	ObS1	VcS1
	Exp.	Lit.														
trans-p-Menth-2-en-1-ol*	1146	1137[b]	-	-	-	-	-	-	-	-	-	-	-	0.5	-	-
Citronellal*	1152	1153[a]	0.7	1.1	-	-	-	-	-	-	-	-	-	-	-	-
cis-Isocitral	1164	1164[a]	1.6	2.1	-	-	-	-	-	-	-	-	-	-	-	-
Borneol*	1172	1169[a]	-	-	-	1.3	-	-	-	-	-	-	-	-	-	-
Isogeranial	1180	1185[a]	2.1	2.8	-	-	-	-	-	-	-	-	-	-	-	-
Terpinen-4-ol*	1186	1177[a]	-	-	-	-	0.9	0.5	0.8	-	0.8	-	0.1	0.3	-	-
cis-Dihydrocarvone	1200	1192[a]	-	-	0.4	2.0	-	-	-	-	-	-	-	-	-	-
Estragole*	1203	1196[a]	-	-	-	-	-	-	-	-	-	-	-	-	18.6	-
trans-Dihydrocarvone	1208	1200[a]	-	-	0.5	3.4	-	-	-	-	-	-	-	-	-	-
Nerol*	1229	1229[a]	2.5	2.2	-	-	-	-	-	-	-	-	-	-	-	-
Thymol-methyl-ether	1230	1235[a]	-	-	-	-	0.8	1.9	1.3	-	1.8	-	-	-	-	-
cis-Carveol	1231	1229[a]	-	-	0.1	-	-	-	-	-	-	-	-	-	-	-
neo-iso-Dihydrocarveol	1242	1228[a]	-	-	0.1	0.8	-	-	-	-	-	-	-	-	-	-
Ascaridole	1246	1237[c]	-	-	-	-	-	-	-	-	-	0.4	0.4	-	-	-
Neral*	1246	1252[b]	11.9	18.1	-	-	-	-	-	-	-	-	-	-	-	-
Carvone*	1259	1258[c]	-	-	12.2	78.2	-	-	-	-	-	-	-	-	-	-
Geraniol*	1260	1240[b]	19	8.1	-	-	-	-	-	-	-	-	-	-	-	-
Piperitone*	1265	1264[c]	-	-	-	4.8	-	-	-	-	-	-	-	14.8	-	-
Geranial*	1272	1270[b]	24.5	24.8	-	-	-	-	-	-	-	-	-	-	-	-
Bornyl acetate	1289	1285[a]	-	-	-	-	-	-	-	-	-	-	-	-	-	0.9
Thymol*	1290	1290[a]	-	-	-	-	14.0	8.0	75.3	82.9	49.4	-	0.2	-	-	-
Carvacrol*	1300	1298[a]	-	-	-	-	0.9	35	4.9	1.2	2.7	-	0.4	-	-	-
iso-Ascaridole	1310	1307[c]	-	-	-	-	-	-	-	-	-	0.3	-	-	-	-
trans-Carvyl acetate	1346	1342[a]	-	-	-	0.1	-	-	-	-	-	-	-	-	-	-
Thymyl acetate*	1346	1352[a]	-	-	-	-	-	-	1.6	-	0.5	-	-	-	-	-

Table S1. Cont.

Compound	LRIs (DB-5MS column) Exp.	Lit.	LaCi	LaCif	LaCaf1	LaCaf2	LoP	LoC	LoT	LoTf	LoTC	TdS1	TdS2	PaS1	ObS1	VcS1
Piperitenone	1347	1343[a]	-	-	0.3	0.3	-	-	-	-	-	-	-	14.8	-	-
α-Cubebene	1355	1351[a]	-	-	-	-	-	-	-	-	-	-	-	0.8	-	-
Carvacryl acetate	1366	1372[a]	-	-	-	-	-	2.0	-	-	-	-	-	-	-	-
Neryl acetate	1376	1361[a]	0.4	0.6	-	-	-	-	-	-	-	-	-	-	-	-
α-Ylangene	1380	1375[a]	-	-	-	-	-	-	-	-	-	-	-	-	-	0.6
Cyclosativene	1382	1374[c]	-	-	-	-	-	-	-	-	-	-	-	1.6	-	-
Isoledene	1382	1376[a]	-	-	-	-	-	-	-	-	-	-	-	-	-	0.5
Geranyl acetate*	1383	1381[a]	2.8	4.8	-	-	-	-	-	-	-	-	-	-	-	-
α-Copaene	1385	1376[a]	-	-	-	0.3	0.6	0.7	-	-	-	-	-	2.9	-	7.0
β-Bourbonene	1396	1387[a]	-	-	0.3	3.8	-	-	-	-	-	-	-	-	0.6	1.1
β-Elemene	1398	1390[a]	2.6	1.2	0.1	1.5	-	-	-	-	-	4.0	5.0	0.7	2.2	2.8
α-Gurjunene	1419	1409[b]	-	-	-	-	-	-	-	-	-	-	-	1.3	-	-
α-Santalene	1428	1420[c]	-	-	-	-	-	-	-	-	-	-	-	0.8	-	-
β-Copaene	1433	1432[a]	-	-	-	0.6	-	-	-	-	-	-	-	-	-	-
trans-β-Caryophyllene*	1433	1427[c]	9.1	13.3	-	0.1	15.1	4.4	5.4	7.0	1.6	4.0	4.9	7.4	0.9	19.2
β-Gurjunene	1437	1432[a]	-	-	-	0.4	-	0.4	-	-	-	-	-	-	-	-
trans-α-Bergamotene	1440	1434[a]	-	-	-	-	0.5	0.3	1.0	-	-	-	-	-	1.1	-
α-Guaiene	1443	1437[a]	-	2.9	-	-	-	-	-	-	-	-	-	-	1.0	-
6,9-Guaiadiene	1451	1444[a]	-	-	-	-	-	-	-	-	-	1.3	1.8	-	-	1.6
Aromadendrene	1452	1441[b]	-	-	-	-	-	-	-	-	-	-	-	1.4	-	-
trans-β-Farnesene	1460	1456[a]	0.5	0.1	-	0.2	-	-	-	-	-	-	-	0.6	-	-
N.I. $M^{+\bullet}$ *m/z* 204	1462	-	-	-	-	-	-	-	-	-	-	-	-	-	-	1.0
α-Humulene*	1467	1468[c]	2.8	2.8	-	-	8.1	1.1	3.2	-	0.9	-	0.4	1.5	2.5	2.7
γ-Muurolene	1473	1479[a]	-	-	-	-	0.6	-	-	-	-	-	-	1.5	-	0.6
trans-Muurola-4(14),5-diene	1475	1493[a]	-	-	-	-	-	-	-	-	-	-	-	-	0.7	-

Table S1. Cont.

Compound	LRIs (DB-5MS column)	LaCi	LaCif	LaCaf1	LaCaf2	LoP	LoC	LoT	LoTf	LoTC	TdS1	TdS2	PaS1	ObS1	VcS1

Compound	LRIs (DB-5MS column)		LaCi	LaCif	LaCaf1	LaCaf2	LoP	LoC	LoT	LoTf	LoTC	TdS1	TdS2	PaS1	ObS1	VcS1
	Exp.	Lit.														
trans-9-*epi*-Caryophyllene	1477	1466[a]	-	-	-	0.3	-	-	-	-	-	-	-	-	-	-
N.I. $M^{+\bullet}$ *m/z* 180	1477	-	-	-	-	-	-	0.4	-	-	-	-	-	-	-	-
trans-Cadina-1(6),4-diene	1482	1476[a]	-	-	-	-	-	-	-	-	-	-	-	1.1	-	-
Aristolochene	1483	1488[a]	-	-	-	-	-	-	-	-	-	17.9	20.9	-	-	-
N.I. $M^{+\bullet}$ *m/z* 180	1484	-	-	-	-	-	-	6.1	-	-	-	-	-	-	-	-
Germacrene D*	1492	1481[a]	4.3	1.5	0.1	-	0.9	-	-	-	-	-	-	1.7	4.9	12.3
Premnaspirodiene	1499	1505[a]	-	-	-	-	-	-	-	-	-	3.7	4.7	-	-	-
β-Selinene	1502	1490[a]	-	-	-	-	0.5	0.3	-	-	-	5.2	5.8	-	-	-
Valencene	1503	1496[a]	-	-	-	-	-	-	-	-	-	7.4	6.5	1.2	-	-
Viridiflorene	1503	1496[a]	-	-	-	-	-	-	-	-	-	-	-	-	-	2.4
Viridiflorol	1503	1496[a]	-	-	-	-	-	-	-	-	-	-	-	6.5	-	1.6
α-Muurolene	1506	1500[a]	-	-	-	-	0.9	-	-	-	-	-	-	-	-	0.6
Geranyl isobutyrate	1506	1515[a]	1.2	0.3	-	-	-	-	-	-	-	-	-	-	-	-
Bicyclogermacrene	1509	1500[a]	-	-	-	-	-	-	-	-	-	-	-	2.8	2.2	3.9
α-Selinene	1509	1498[a]	-	-	-	-	-	0.8	-	-	-	1.6	1.7	-	-	-
α-Bulnesene	1510	1509[a]	-	0.6	-	-	-	-	-	-	-	-	-	-	1.1	-
δ-Amorphene	1515	1511[c]	-	-	-	-	-	-	-	-	-	-	-	1.0	-	-
trans-β-Guaiene	1517	1502[a]	2.2	-	-	-	-	-	-	-	-	-	-	-	-	11.8
β-Bisabolene	1517	1505[a]	-	-	-	-	0.8	0.8	0.8	-	-	-	-	-	-	-
γ-Cadinene	1523	1513[a]	-	-	-	-	0.5	0.3	-	-	-	-	-	0.7	3.7	-
δ-Cadinene	1528	1523[a]	-	-	-	-	1.2	1.1	-	-	-	-	0.3	5.5	-	2.8
cis-Calamenene	1530	1529[a]	-	-	-	-	0.5	-	-	-	-	-	-	-	0.4	-
trans-γ-Bisabolene	1533	1531[a]	-	-	-	-	-	0.4	-	-	-	-	-	-	-	-
7-*epi*-α-Selinene	1533	1534[c]	-	-	-	-	-	-	-	-	-	0.5	0.6	-	-	-
Zonarene	1534	1529[a]	-	-	-	-	-	-	-	-	-	-	-	1.2	-	-
α-Cadinene	1535	1538[a]	-	-	-	-	-	-	-	-	-	-	-	0.6	-	0.6

Table S1. Cont.

Compound	LRIs (DB-5MS column)		LaCi	LaCif	LaCaf1	LaCaf2	LoP	LoC	LoT	LoTf	LoTC	TdS1	TdS2	PaS1	ObS1	VcS1
	Exp.	Lit.														
N.I. $M^{+\bullet}$ *m/z* 222	1539	-	-	-	-	-	-	0.2	-	-	-	-	-	-	-	-
N.I. $M^{+\bullet}$ *m/z* 204	1544	-	-	-	-	-	-	0.4	-	-	-	-	-	-	-	-

trans-α-Bisabolene	1545	1544[c]	2.0	0.3	-	-	-	-	-	-	-	-	-	-	-	-
Selina-3,7(11)-diene	1552	1546[a]	-	-	-	-	-	-	-	-	-	-	-	-	-	1.0
N.I. $M^{+\bullet}$ *m/z* 222	1554	-	-	-	-	-	-	1.7	-	-	-	-	-	-	-	-
Elemol	1556	1548[a]	-	-	-	-	-	-	-	-	-	0.2	0.3	-	-	-
N.I. $M^{+\bullet}$ *m/z* 204	1557	-	-	-	-	-	-	-	-	-	-	-	-	-	-	0.8
trans-Nerolidol	1562	1563[a]	-	-	-	-	0.9	0.7	-	-	-	-	-	-	0.5	1.3
Zierone	1564	1574[c]	-	-	-	-	-	-	-	-	-	-	0.3	-	-	-
Germacren D-4-ol	1585	1575[c]	-	-	-	-	-	-	-	-	-	-	-	0.4	-	-
Spathulenol	1591	1578[a]	-	-	-	-	-	-	-	-	-	-	-	1.9	0.8	-
Caryophyllene oxide*	1595	1583[a]	1.8	0.1	-	-	2.5	0.9	1.3	-	0.4	3.3	3.2	3.8	-	0.6
Guaiol	1606	1600[a]	-	-	-	-	0.6	-	-	-	-	3.5	3.3	-	-	0.7
Humulene epoxide II	1625	1608[a]	-	-	-	-	1.0	-	0.5	-	-	-	-	-	-	-
1,10-di-*epi*-Cubenol	1629	1619[a]	-	-	-	-	-	-	-	-	-	-	-	-	0.9	-
neo-Intermedeol	1632	1631[c]	-	-	-	-	-	-	-	-	-	1.3	1.2	-	-	-
epi-α-Cadinol	1640	1640[c]	-	-	-	-	-	0.9	-	-	-	-	-	0.8	4.2	-
lntermedeol	1640	1658[a]	-	-	-	-	-	-	-	-	-	-	-	-	-	0.6
γ-Eudesmol	1644	1632[a]	-	-	-	-	1.0	0.2	-	-	-	-	-	-	-	-
N.I. $M^{+\bullet}$ *m/z* 220 + *m/z* 204	1647	-	-	-	-	-	0.5	-	-	-	-	-	-	-	-	-
epi-α-Muurolol	1655	1642[c]	-	-	-	-	-	-	-	-	-	-	-	0.2	-	-
Eudesm-7(11)-en-4-ol	1696	1700[a]	-	-	-	-	-	-	-	-	-	1.2	1.0	-	-	-
α-Eudesmol	1670	1653[a]	-	-	-	-	2.6	0.3	-	-	-	-	-	0.8	-	-
Pogostol	1671	1660[c]	-	-	-	-	-	-	-	-	-	2.8	3.3	-	-	-
α-Selin-11-en-4-ol	1674	1659[a]	-	-	-	-	-	-	-	-	-	-	-	-	-	0.5

Table S1. Cont.

Compound	LRIs (DB-5MS column)		LaCi	LaCif	LaCaf1	LaCaf2	LoP	LoC	LoT	LoTf	LoTC	TdS1	TdS2	PaS1	ObS1	VcS1
	Exp.	Lit.														
Germacra-4,5,10-trien-1-α-ol	1680	1686[a]	-	-	-	-	-	-	-	-	-	3.5	3.6	-	-	-
Valerenal	1720	1716[c]	-	-	-	-	-	-	-	-	-	0.6	0.6	-	-	-
Vetiselinenol	1740	1730[a]	-	-	-	-	-	-	-	-	-	2.1	1.7	-	-	-

Dehydrofukinone	1827	1820[c]	-	-	-	-	-	-	-	-	-	25.4	19.3	-	-	-
trans,*trans*-Geranil linalool	2028	2026[a]	-	-	-	-	-	-	-	-	-	-	-	-	-	1.6

LRIs, linear retention indices calculated using *n*-alkanes C_8-C_{25} mixture on the DB-5MS (non-polar) column. *L. alba*: neat EO (LaCif) and light fraction (LaCif) of citral chemotype EO; limonene-rich fraction (LaCaf1) and carvone-rich fraction (LaCaf2) of carvone chemotype. *L. origanoides*: neat EO of phellandrene chemotype (LoP), carvacrol (LoC), thymol-carvacrol (LoTC), and thymol (LoT) chemotype; and thymol-rich fraction (LoTf) of thymol chemotype. Neat EOs of *T. diffusa* collected in 2019 (TdS1) and in 2016 (TdS2). Neat EOs of *O. basilicum* (ObS1), *P. auduncum* (*Pa*S1) and *V. curassavica* (VcS1). Exp. Experimental. Lit. Literature [a]Adams, 2007; [b] Babushok et al., 2011; [c] NIST 2017. * Standard compounds were used for confirmatory identification.

Table S2. Docking energies (kcal/mol) of essential oil terpenes with dengue virus serotype 2 proteins

Compound	Structural DENV-2 proteins		
	E	C	prM
Epigallocatechin gallate: positive control	-7.85 ± 0.3	-7.67 ± 0.4	-7.37 ± 0.1
1,10-di-*epi*-Cubenol	-6.02 ± 0.7	-6.46 ± 0.4	-5.12 ± 0.2
1,8-Cineol	-4.76 ± 0.2	-5.07 ± 0.2	-4.24 ± 0.2
6,9-Guaiadiene	-7.30 ± 1.3	-6.69 ± 0.1	-5.15 ± 0.3
7-*epi*-α-Selinene	-6.74 ± 1.0	-6.52 ± 0.3	-5.33 ± 0.1
Eudesm-7(11)-en-4-ol	-6.91 ± 0.4	-6.40 ± 0.3	-5.38 ± 0.2
Aristolochene	-6.60 ± 0.5	-6.66 ± 0.3	-5.41 ± 0.3
Aromadendrene	-6.32 ± 0.9	-6.53 ± 0.3	-5.26 ± 0.1
Ascaridole	-6.12 ± 0.4	-5.67 ± 0.1	-4.88 ± 0.1
Bicyclogermacrene	-6.29 ± 0.5	-6.60 ± 0.3	-5.14 ± 0.1
Borneol	-4.77 ± 0.2	-4.54 ± 0.3	-4.17 ± 0.1
Bornyl acetate	-5.04 ± 0.2	-5.29 ± 0.3	-4.56 ± 0.1
Camphene	-5.55 ± 0.3	-5.41 ± 0.2	-4.14 ± 0.1
Carvacrol	-7.31 ± 0.4	-6.08 ± 0.5	-5.29 ± 0.4
Carvacryl acetate	-7.52 ± 0.8	-5.90 ± 0.2	-5.29 ± 0.3
Carvone	-7.29 ± 0.9	-5.99 ± 0.5	-4.90 ± 0.3
Caryophyllene oxide	-5.62 ± 0.3	-6.46 ± 0.4	-5.26 ± 0.1
cis-Calamenene	-8.73 ± 0.8	-6.91 ± 0.2	-5.33 ± 0.1
cis-Carveol	-6.98 ± 0.4	-5.78 ± 0.4	-4.77 ± 0.3
cis-Dihydrocarvone	-6.48 ± 0.8	-5.64 ± 0.3	-4.51 ± 0.1
cis-Isocitral	-6.08 ± 0.6	-5.37 ± 0.1	-4.78 ± 0.2
cis-Limonene oxide	-6.71 ± 0.5	-5.65 ± 0.5	-4.74 ± 0.3
cis-p-Menth-2-en-1-ol	-6.17 ± 0.5	-5.64 ± 0.2	-4.64 ± 0.1
cis-Sabinene hydrate	-5.93 ± 0.5	-5.81 ± 0.5	-4.21 ± 0.3
cis-β-Ocimene	-6.29 ± 0.5	-5.50 ± 0.2	-4.41 ± 0.1
Citronellal	-5.94 ± 0.3	-5.08 ± 0.3	-4.03 ± 0.3
Cyclosativene	-5.98 ± 0.4	-6.39 ± 0.3	-5.22 ± 0.1
Dehydrofukinone	-6.39 ± 0.8	-6.60 ± 0.5	-5.52 ± 0.3
Elemol	-5.92 ± 0.5	-6.04 ± 0.2	-5.01 ± 0.1
epi-α-Cadinol	-6.38 ± 0.7	-6.50 ± 0.3	-5.29 ± 0.2
epi-α-Muurolol	-6.10 ± 0.6	-6.22 ± 0.4	-5.04 ± 0.1
Estragole	-6.69 ± 0.6	-5.28 ± 0.1	-4.79 ± 0.3
Geranial	-6.32 ± 0.3	-5.22 ± 0.2	-4.29 ± 0.3
Geraniol	-6.14 ± 0.4	-5.24 ± 0.2	-4.24 ± 0.3
Geranyl acetate	-5.97 ± 0.7	-5.69 ± 0.2	-4.87 ± 0.2

Table S2. Cont.

Compound	E	C	prM
Geranyl isobutyrate	-6.07 ± 0.6	-6.15 ± 0.1	-5.10 ± 0.1
Germacra-4,5,10-trien-1-α-ol	-5.90 ± 0.6	-6.34 ± 0.2	-5.35 ± 0.2
Germacren D-4-ol	-6.01 ± 0.7	-6.37 ± 0.3	-5.23 ± 0.2
Germacrene D	-6.79 ± 1.1	-6.49 ± 0.2	-5.36 ± 0.1
Guaiol	-6.30 ± 0.7	-6.85 ± 0.4	-5.41 ± 0.1
Humulene epoxide II	-5.64 ± 0.3	-6.20 ± 0.4	-5.29 ± 0.1
iso-Ascaridole	-6.31 ± 0.5	-5.52 ± 0.4	-4.64 ± 0.2
Isogeranial	-6.13 ± 0.5	-5.34 ± 0.1	-4.74 ± 0.2
Limonene	-7.24 ± 0.1	-5.76 ± 0.6	-4.60 ± 0.4
Linalool	-6.06 ± 0.4	-4.87 ± 0.2	-4.31 ± 0.2
lntermedeol	-5.77 ± 0.4	-6.59 ± 0.3	-5.25 ± 0.0
lsoledene	-6.72 ± 0.7	-6.74 ± 0.3	-5.29 ± 0.1
neo-Intermedeol	-6.34 ± 1.0	-6.41 ± 0.3	-5.19 ± 0.1
neo-*iso*-Dihydrocarveol	-5.04 ± 0.5	-4.94 ± 0.3	-4.15 ± 0.1
Neral	-6.16 ± 0.5	-5.04 ± 0.2	-4.21 ± 0.2
Nerol	-6.13 ± 0.4	-5.27 ± 0.3	-4.42 ± 0.2
Neryl acetate	-6.32 ± 0.5	-5.91 ± 0.1	-5.16 ± 0.2
p-Cymene	-7.27 ± 0.3	-5.81 ± 0.5	-4.89 ± 0.4
Piperitenone	-6.81 ± 0.7	-6.01 ± 0.3	-5.07 ± 0.4
Piperitone	-6.82 ± 0.5	-6.02 ± 0.4	-4.65 ± 0.2
Pogostol	-5.90 ± 0.4	-6.40 ± 0.2	-5.30 ± 0.3
Premnaspirodiene	-5.75 ± 0.4	-6.44 ± 0.4	-5.07 ± 0.2
Sabinene	-6.59 ± 0.3	-5.86 ± 0.6	-4.62 ± 0.3
Selina-3,7(11)-diene	-6.61 ± 1.0	-6.81 ± 0.3	-5.18 ± 0.1
Spathulenol	-6.17 ± 0.6	-6.29 ± 0.3	-5.49 ± 0.2
Terpinen-4-ol	-6.38 ± 0.4	-5.72 ± 0.6	-4.81 ± 0.4
Terpinolene	-7.03 ± 0.6	-5.74 ± 0.5	-4.89 ± 0.4
Thymol	-6.98 ± 0.5	-5.98 ± 0.5	-5.31 ± 0.3
Thymol-methyl-ether	-7.11 ± 0.4	-5.64 ± 0.2	-4.99 ± 0.3
Thymyl acetate	-7.01 ± 0.8	-5.88 ± 0.2	-5.19 ± 0.1
trans, *trans*-Geranil linalool	-6.37 ± 0.3	-6.61 ± 0.1	-5.31 ± 0.2
trans-9-*epi*-Caryophyllene	-5.60 ± 0.6	-6.08 ± 0.3	-5.32 ± 0.2
trans-Cadina-1(6),4-diene	-7.23 ± 1.2	-6.74 ± 0.4	-5.11 ± 0.0
trans-Carvyl acetate	-6.84 ± 1.0	-6.03 ± 0.1	-4.92 ± 0.2
trans-Dihydrocarvone	-7.23 ± 0.3	-5.85 ± 0.3	-4.84 ± 0.2
trans-Muurola-4(14),5-diene	-6.93 ± 1.4	-6.81 ± 0.4	-5.12 ± 0.4
trans-Nerolidol	-5.87 ± 0.4	-5.80 ± 0.2	-4.63 ± 0.2

Table S2. Cont.

Compound	E	C	prM

trans-*p*-Menth-2-en-1-ol	-6.53 ± 0.5	-5.58 ± 0.1	-4.60 ± 0.1
trans-*p*-Mentha-2,8-dien-1-ol	-6.14 ± 0.4	-5.59 ± 0.2	-4.50 ± 0.2
trans-α-Bergamotene	-6.07 ± 0.3	-6.32 ± 0.3	-4.97 ± 0.1
trans-α-Bisabolene	-6.91 ± 0.6	-6.65 ± 0.1	-5.50 ± 0.0
trans-β-Caryophyllene	-6.02 ± 0.4	-6.63 ± 0.4	-5.36 ± 0.2
trans-β-Farnesene	-6.43 ± 0.7	-6.04 ± 0.1	-5.01 ± 0.4
trans-β-Guaiene	-6.18 ± 1.0	-6.70 ± 0.3	-5.36 ± 0.3
trans-β-Ocimene	-6.49 ± 0.2	-5.86 ± 0.3	-4.45 ± 0.2
trans-γ-Bisabolene	-6.97 ± 0.7	-6.28 ± 0.1	-5.01 ± 0.2
Valencene	-6.84 ± 0.9	-6.50 ± 0.3	-5.34 ± 0.2
Valerenal	-6.01 ± 0.6	-6.58 ± 0.2	-5.26 ± 0.2
Vetiselinenol	-7.07 ± 0.8	-6.59 ± 0.3	-5.45 ± 0.1
Viridiflorene	-8.13 ± 0.9	-6.61 ± 0.2	-5.42 ± 0.1
Viridiflorol	-6.24 ± 0.5	-6.09 ± 0.2	-5.40 ± 0.2
Zierone	-5.95 ± 0.5	-6.53 ± 0.3	-5.20 ± 0.3
Zonarene	-7.46 ± 0.9	-6.54 ± 0.3	-5.10 ± 0.1
α-Bulnesene	-7.42 ± 1.0	-6.87 ± 0.4	-5.42 ± 0.3
α-Cadinene	-8.28 ± 1.1	-6.72 ± 0.4	-5.26 ± 0.1
α-Copaene	-6.29 ± 0.5	-6.60 ± 0.3	-5.14 ± 0.1
α-Cubebene	-7.62 ± 1.0	-6.74 ± 0.3	-5.40 ± 0.2
α-Eudesmol	-6.37 ± 0.8	-6.43 ± 0.4	-5.47 ± 0.3
α-Guaiene	-8.26 ± 1.1	-6.73 ± 0.3	-5.29 ± 0.2
α-Gurjunene	-7.83 ± 1.0	-5.72 ± 0.2	-5.42 ± 0.2
α-Humulene	-5.74 ± 0.7	-6.50 ± 0.4	-5.29 ± 0.3
α-Muurolene	-5.87 ± 0.5	-6.78 ± 0.4	-5.27 ± 0.3
α-Phellandrene	-7.60 ± 0.2	-7.38 ± 0.5	-5.84 ± 0.2
α-Pinene	-5.09 ± 0.3	-5.48 ± 0.3	-4.24 ± 0.1
α-Santalene	-6.11 ± 0.5	-6.34 ± 0.2	-4.75 ± 0.1
α-Selin-11-en-4-ol	-6.75 ± 0.9	-4.53 ± 0.3	-5.26 ± 0.2
α-Selinene	-7.98 ± 1.1	-6.63 ± 0.2	-5.19 ± 0.3
α-Terpinene	-7.10 ± 0.6	-5.78 ± 0.6	-4.96 ± 0.4
α-Terpineol	-6.67 ± 0.4	-5.90 ± 0.5	-4.70 ± 0.3
α-Tujene	-6.16 ± 0.7	-6.06 ± 0.3	-4.69 ± 0.2
α-Ylangene	-6.11 ± 0.3	-6.63 ± 0.3	-5.16 ± 0.3
β-Bisabolene	-7.83 ± 0.5	-6.44 ± 0.2	-5.55 ± 0.2
β-Bourbonene	-7.95 ± 0.9	-6.76 ± 0.6	-5.17 ± 0.2
β-Copaene	-6.22 ± 0.7	-6.57 ± 0.3	-5.18 ± 0.2
β-Elemene	-6.07 ± 1.0	-6.04 ± 0.3	-4.85 ± 0.0

Table S2. Cont.

Compound	E	C	prM

β-Gurjunene	-7.83 ± 1.0	-6.72 ± 0.2	-5.42 ± 0.2
β-Myrcene	-6.12 ± 0.3	-5.08 ± 0.4	-4.08 ± 0.2
β-Phellandrene	-6.99 ± 0.5	-6.03 ± 0.6	-4.92 ± 0.3
β-Pinene	-5.11 ± 0.4	-5.36 ± 0.3	-4.21 ± 0.1
β-Selinene	-7.71 ± 1.2	-6.53 ± 0.3	-5.38 ± 0.1
γ-Cadinene	-8.19 ± 1.0	-6.77 ± 0.2	-5.27 ± 0.3
γ-Eudesmol	-6.30 ± 0.4	-6.70 ± 0.1	-5.43 ± 0.2
γ-Muurolene	-5.87 ± 0.5	-6.79 ± 0.4	-5.27 ± 0.3
γ-Terpinene	-7.28 ± 0.3	-5.79 ± 0.5	-4.96 ± 0.4
Δ3-Carene	-5.86 ± 0.4	-5.86 ± 0.4	-4.27 ± 0.1
δ-Amorphene	-7.96 ± 1.0	-6.80 ± 0.3	-5.25 ± 0.2
δ-Cadinene	-8.41 ± 0.9	-6.67 ± 0.3	-5.47 ± 0.3

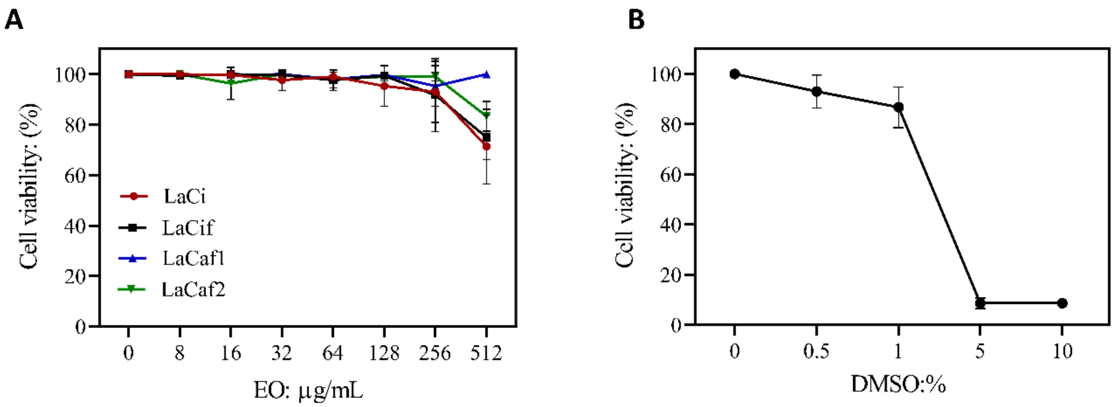

Figure S1. Representative results of the cytotoxicity assay. Essential oils and fractions of *L. alba* citral (LaCi and LaCif) and carvone (LaCaf1 and LaCaf2) chemotypes. Vero cells were cultured for 72 h in the presence of essential oil. Relative cell viability was measured by crystal violet quantification. Data represent averages and ± SD of six independent measurements. Dimethyl sulfoxide (DMSO) is a positive control. The remaining ten essential oils studied (Table 1) showed similar results.
